# Supplementary material for: The role of Nef in the long-term persistence of the replication-competent HIV reservoir in South African women
Source: bioRxiv. 2024 Nov 3:2024.11.01.621615. Preprint. [Version 1] doi: 10.1101/2024.11.01.621615 (PMC11565997; doi:10.1101/2024.11.01.621615)
Supplement: Supplement 1 [file NIHPP2024.11.01.621615v1-supplement-1.pdf]

bioRxiv preprint doi: https://doi.org/10.1101/2024.11.01.621615; this version posted November 3, 2024. The copyright holder for this preprint (which was not certified by peer review) is the author/funder. This article is a US Government work. It is not subject to copyright under 17 USC 105 and is also made available for use under a CC0 license.

Supporting information captions and figures (figure after caption):

Figure S1. Individual participant Nef amino acid trees. OGV *nef* sequences were translated, aligned by Clustal W in MEGA11 [34] and trees were generated using PhyML [52] in DIVEIN [53]. Trees were mid-point rooted and sorted by decreasing node order. A scale bar is included below each tree. Labels indicate the time pre-ART that each OGV was estimated to enter the reservoir in weeks (wks). Red node labels indicate amino acid sequences that were selected for synthesis and subsequent testing for function. Black node labels represent OGV sequences that were not selected for further functional testing in this study.

bioRxiv preprint doi: https://doi.org/10.1101/2024.11.01.621615; this version posted November 3, 2024. The copyright holder for this preprint (which was not certified by peer review) is the author/funder. This article is a US Government work. It is not subject to copyright under 17 USC 105 and is also made available for use under a CC0 license.

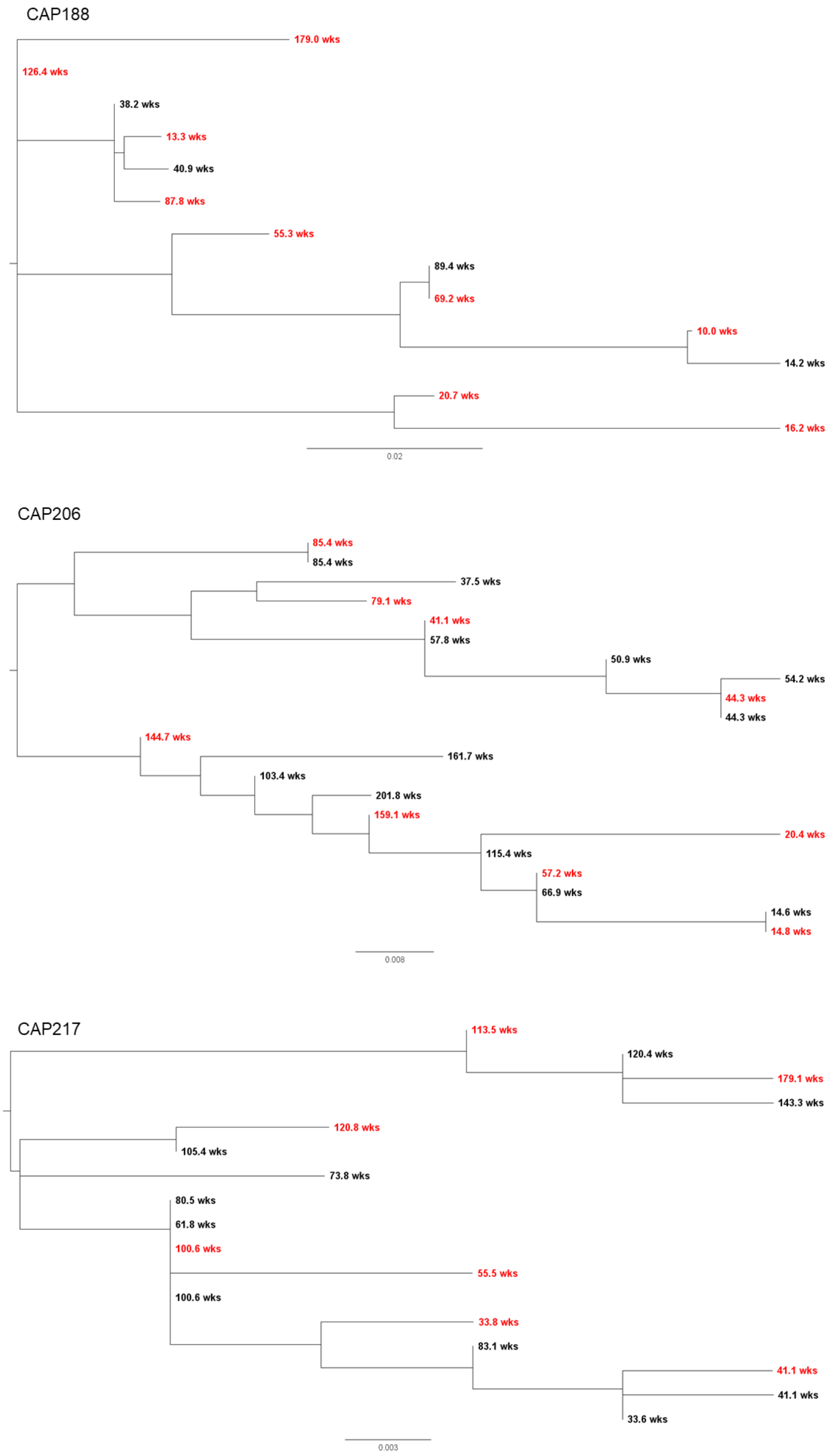

429

bioRxiv preprint doi: https://doi.org/10.1101/2024.11.01.621615; this version posted November 3, 2024. The copyright holder for this preprint (which was not certified by peer review) is the author/funder. This article is a US Government work. It is not subject to copyright under 17 USC 105 and is also made available for use under a CC0 license.

CAP257

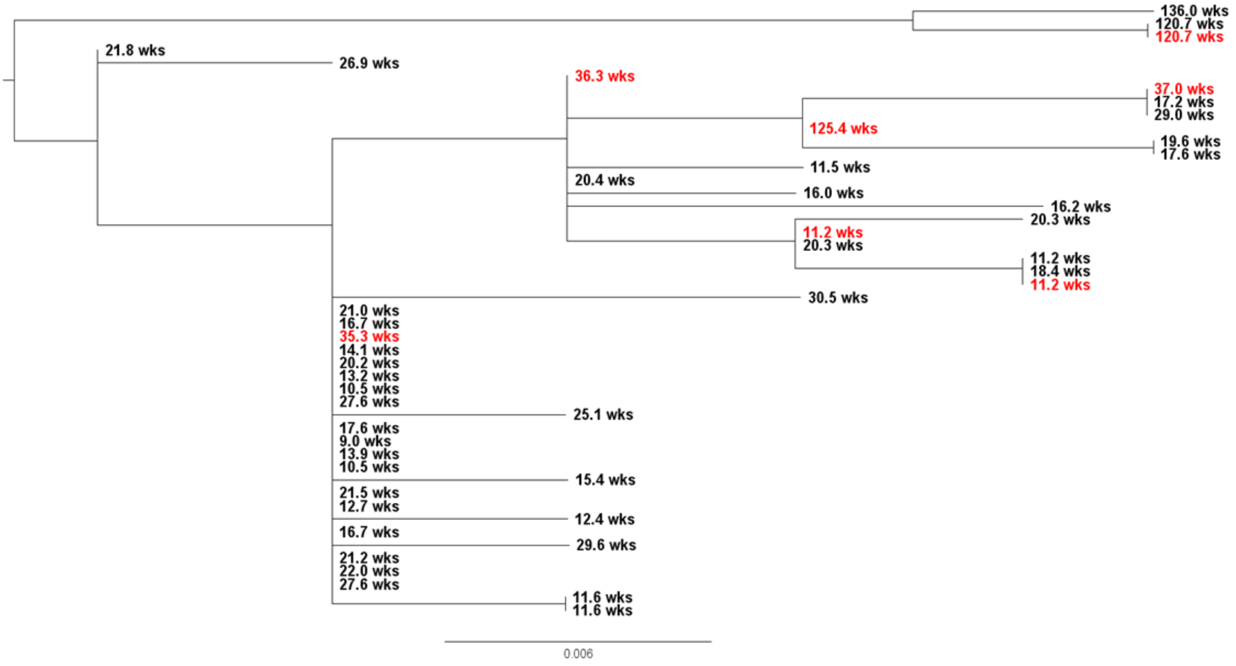

CAP268

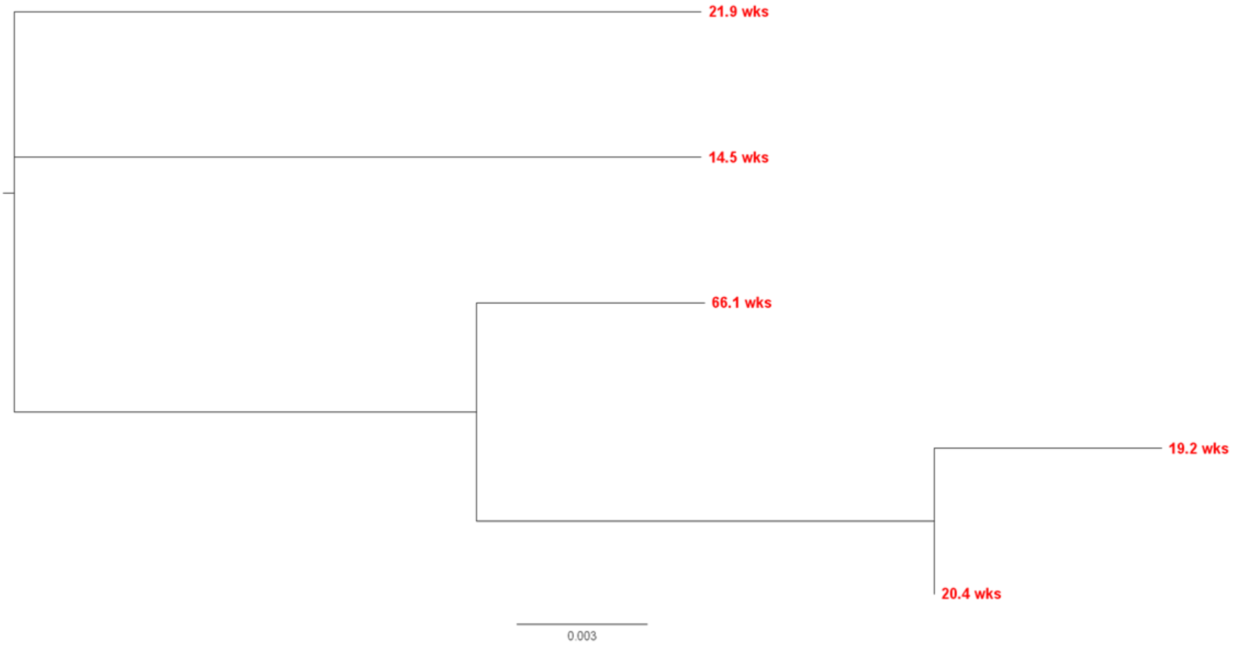

bioRxiv preprint doi: https://doi.org/10.1101/2024.11.01.621615; this version posted November 3, 2024. The copyright holder for this preprint (which was not certified by peer review) is the author/funder. This article is a US Government work. It is not subject to copyright under 17 USC 105 and is also made available for use under a CC0 license.

CAP280

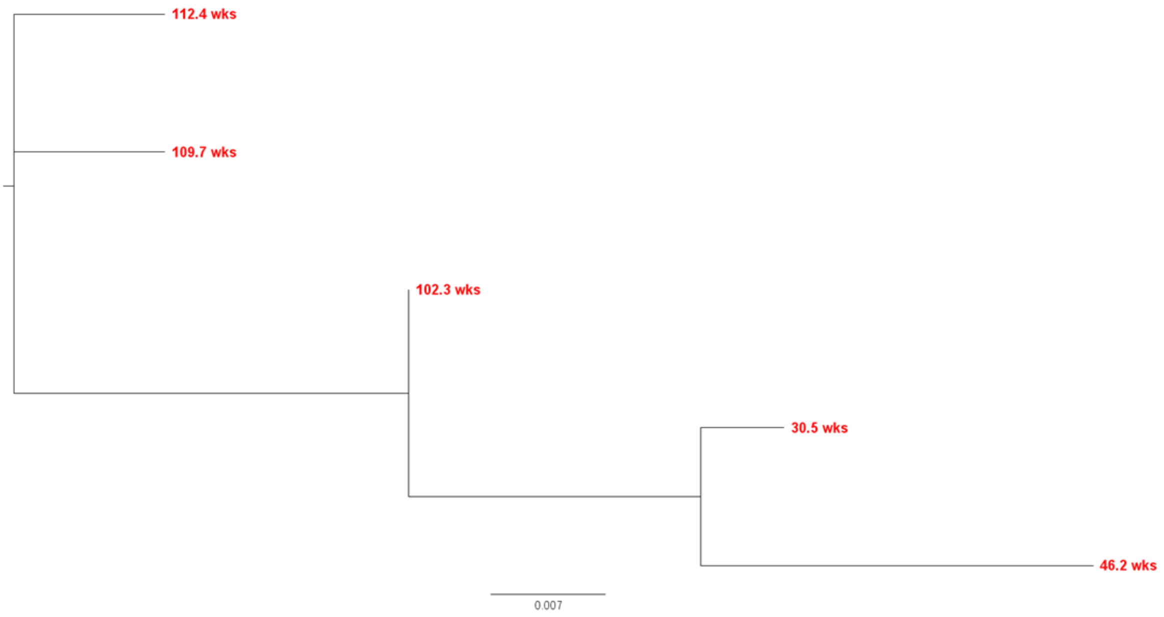

CAP287

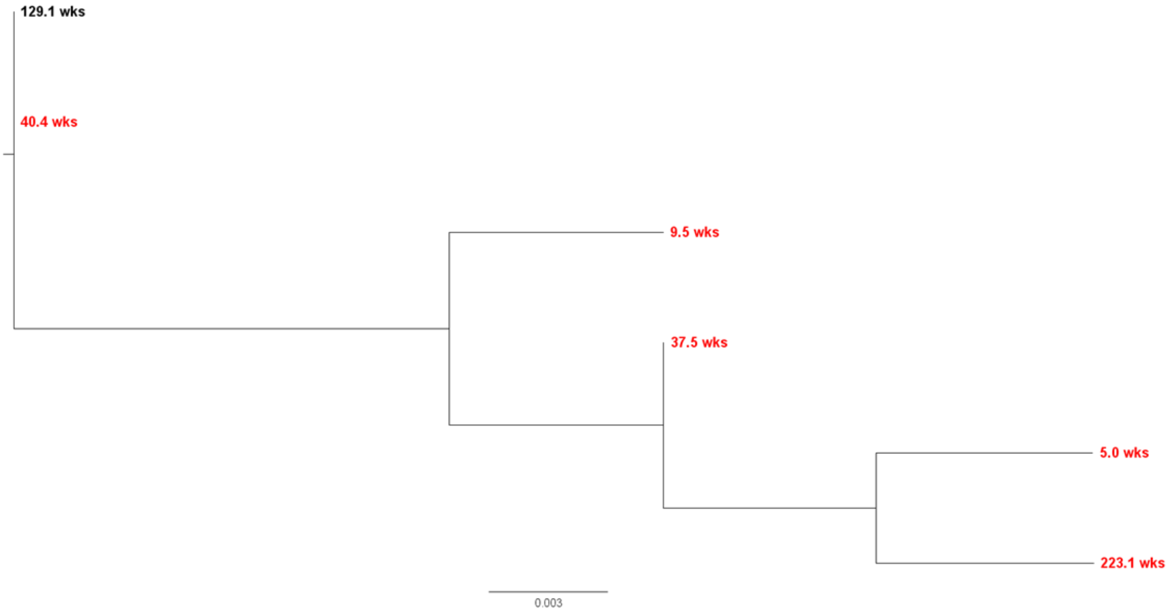

CAP288

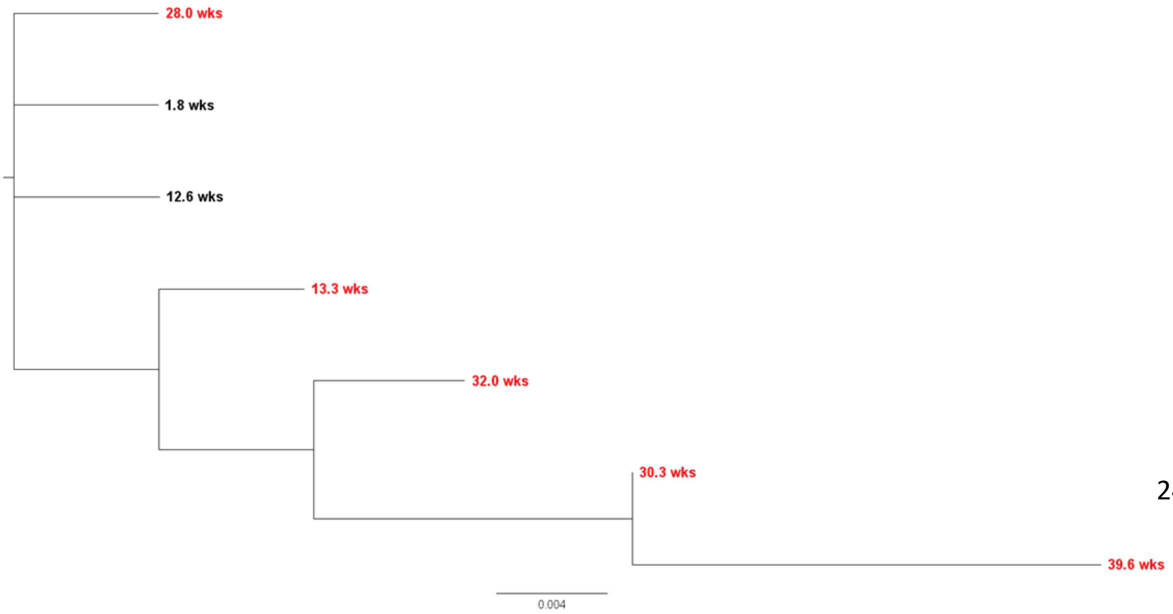

434

bioRxiv preprint doi: https://doi.org/10.1101/2024.11.01.621615; this version posted November 3, 2024. The copyright holder for this preprint (which was not certified by peer review) is the author/funder. This article is a US Government work. It is not subject to copyright under 17 USC 105 and is also made available for use under a CC0 license.

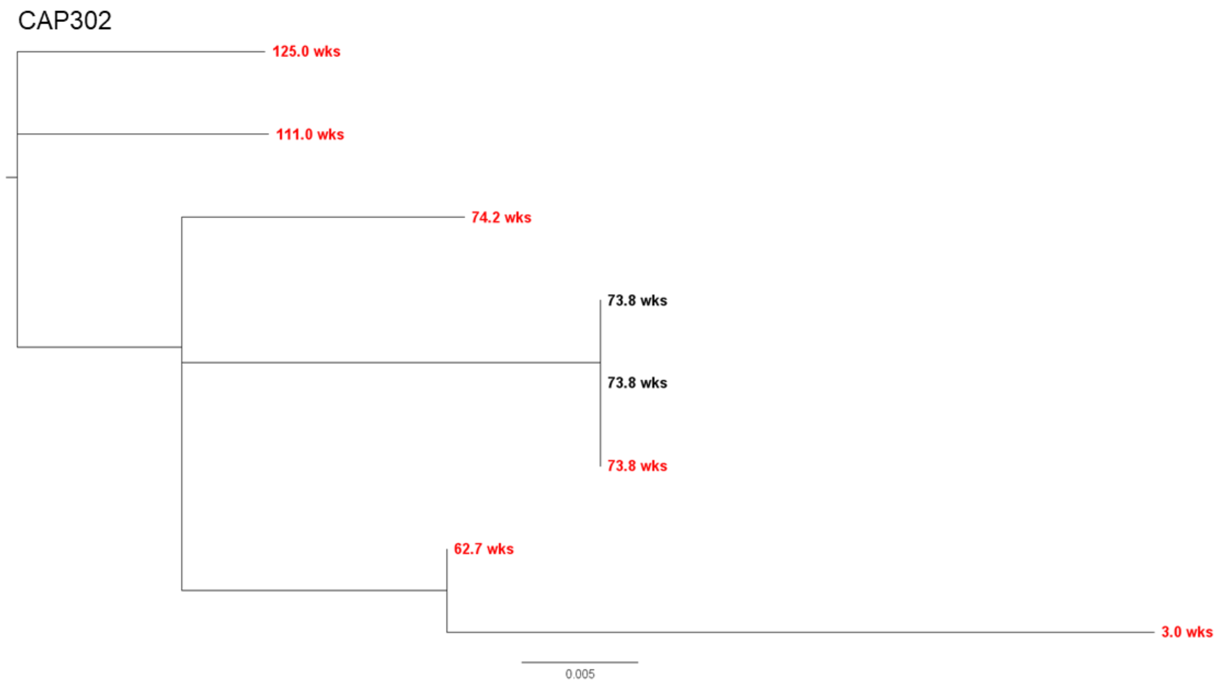

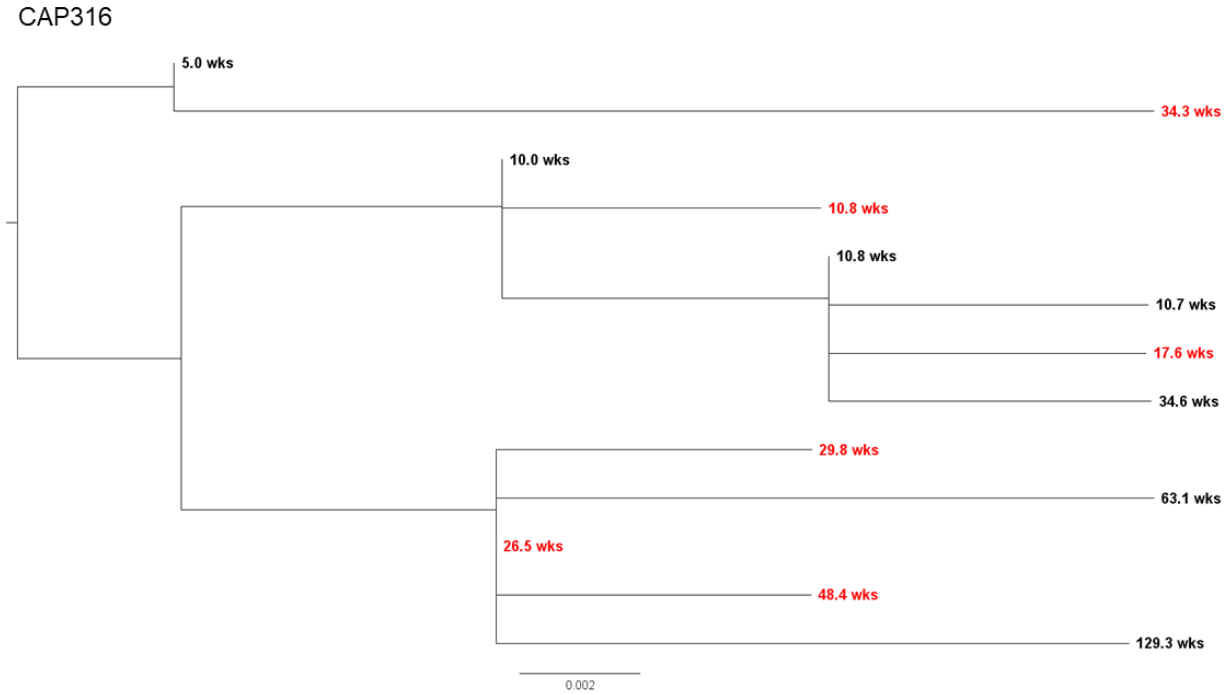

bioRxiv preprint doi: https://doi.org/10.1101/2024.11.01.621615; this version posted November 3, 2024. The copyright holder for this preprint (which was not certified by peer review) is the author/funder. This article is a US Government work. It is not subject to copyright under 17 USC 105 and is also made available for use under a CC0 license.

CAP336

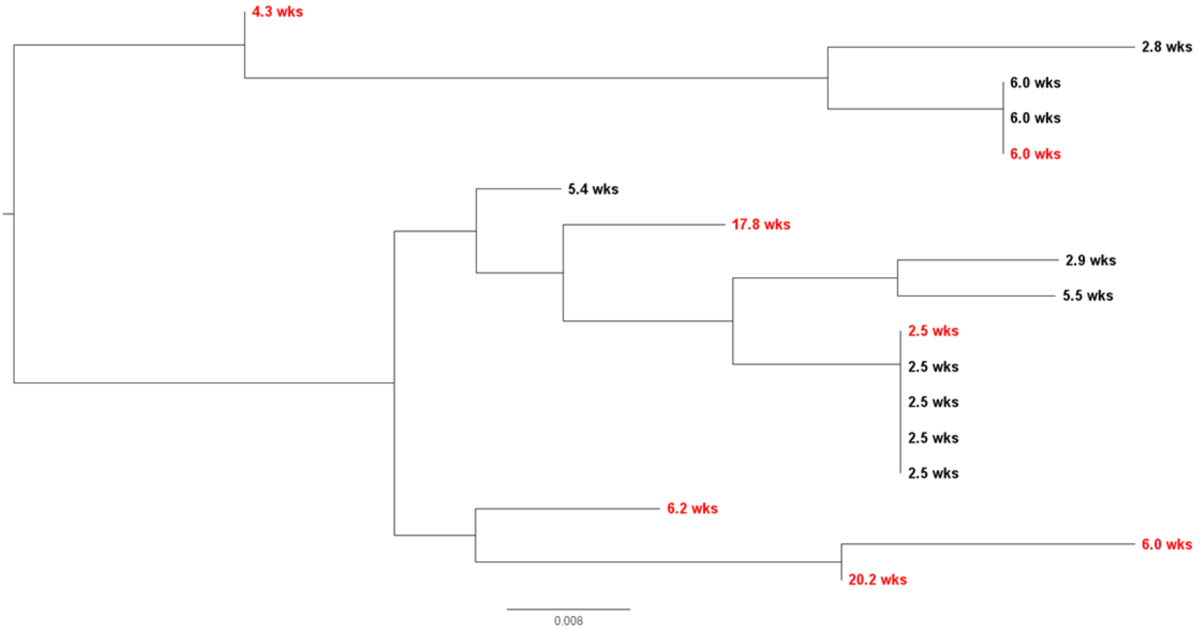

CAP337

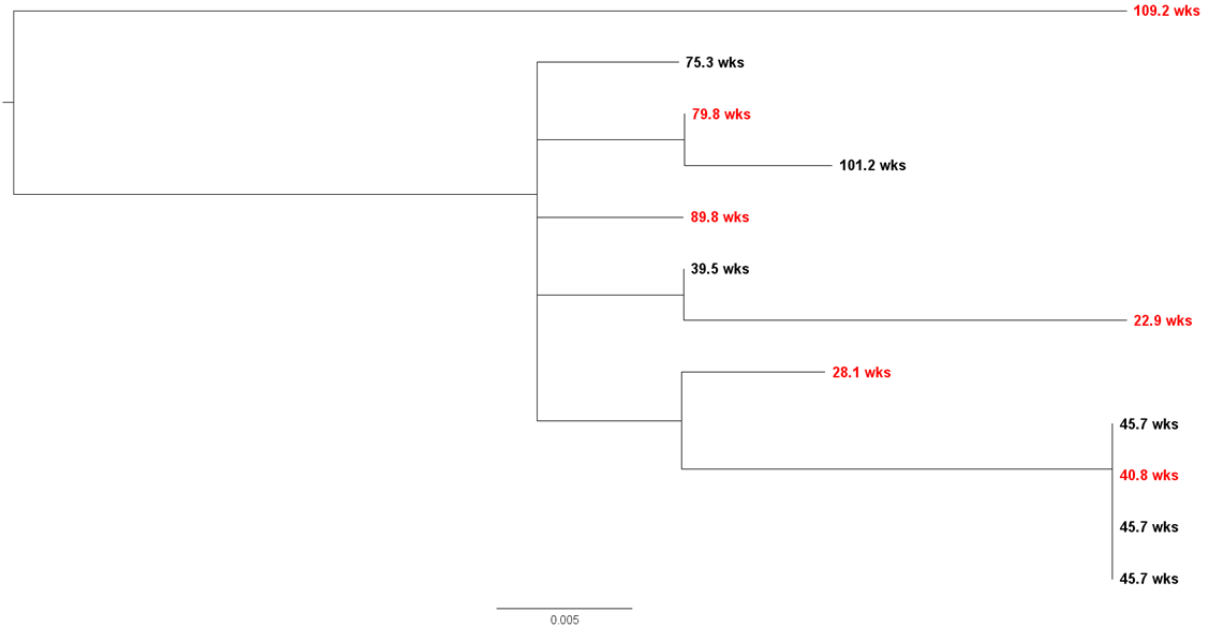

bioRxiv preprint doi: https://doi.org/10.1101/2024.11.01.621615; this version posted November 3, 2024. The copyright holder for this preprint (which was not certified by peer review) is the author/funder. This article is a US Government work. It is not subject to copyright under 17 USC 105 and is also made available for use under a CC0 license.

Figure S2. Gating strategy. Live infected Sup-T1 cells were infected with Nef-pseudotyped viruses. Sup-T1 cells were identified, and doublets were excluded before gating for live cells (gates indicated in purple) which were Near-Infrared$^-$ (viability dye). Subsequently, infected cells were gated as eGFP$^+$ events (red gate) while uninfected cells were identified as eGFP$^-$ (blue gate). In this representative plot, 28.5% of live, single cells were infected. Gates were set based on single stained Sup-T1 cells as well as fluorescence minus one controls (FMOs), stained for all **fluorophores except that which is being gated on. Histogram overlays for either CD4 or MHC-I** expression show the fluorescence intensity shift between infected cells (red peaks) and uninfected cells (blue peaks).

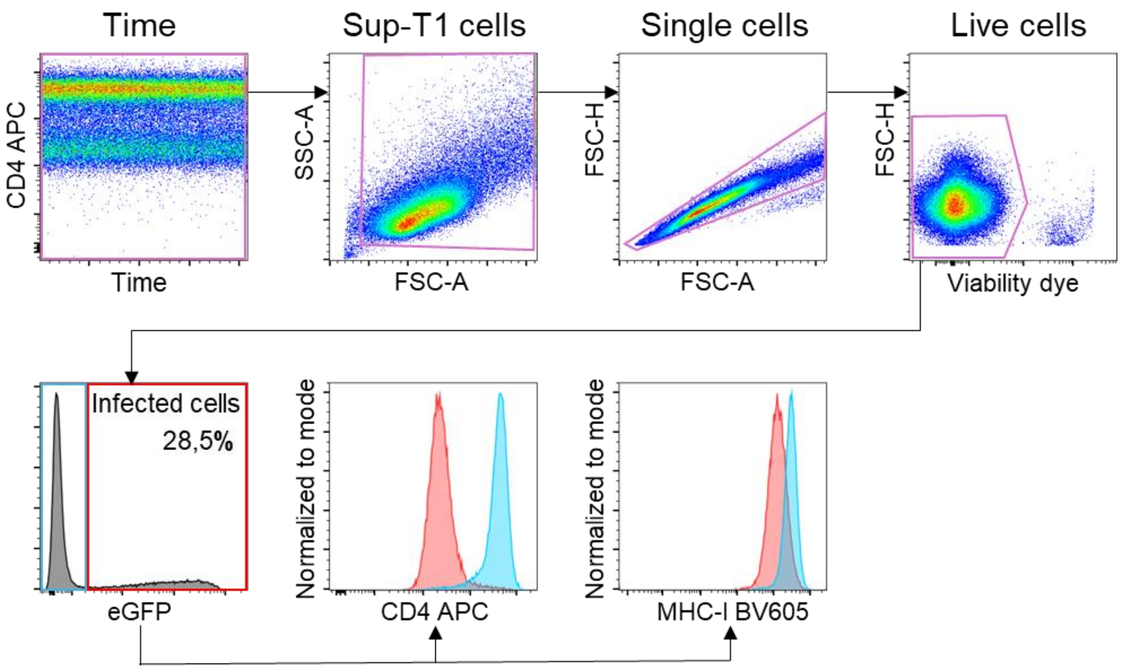

bioRxiv preprint doi: https://doi.org/10.1101/2024.11.01.621615; this version posted November 3, 2024. The copyright holder for this preprint (which was not certified by peer review) is the author/funder. This article is a US Government work. It is not subject to copyright under 17 USC 105 and is also made available for use under a CC0 license.

Figure S3. Participants with a significant linear relationship between MHC-I downregulation activity and proviral survival time. Each point on the graph represents geometric mean MHC-I downregulation for a unique *nef* clone and the error bars represent the 95% CI. Linear regression best fit lines and 95% CI were plotted for significant linear relationships.

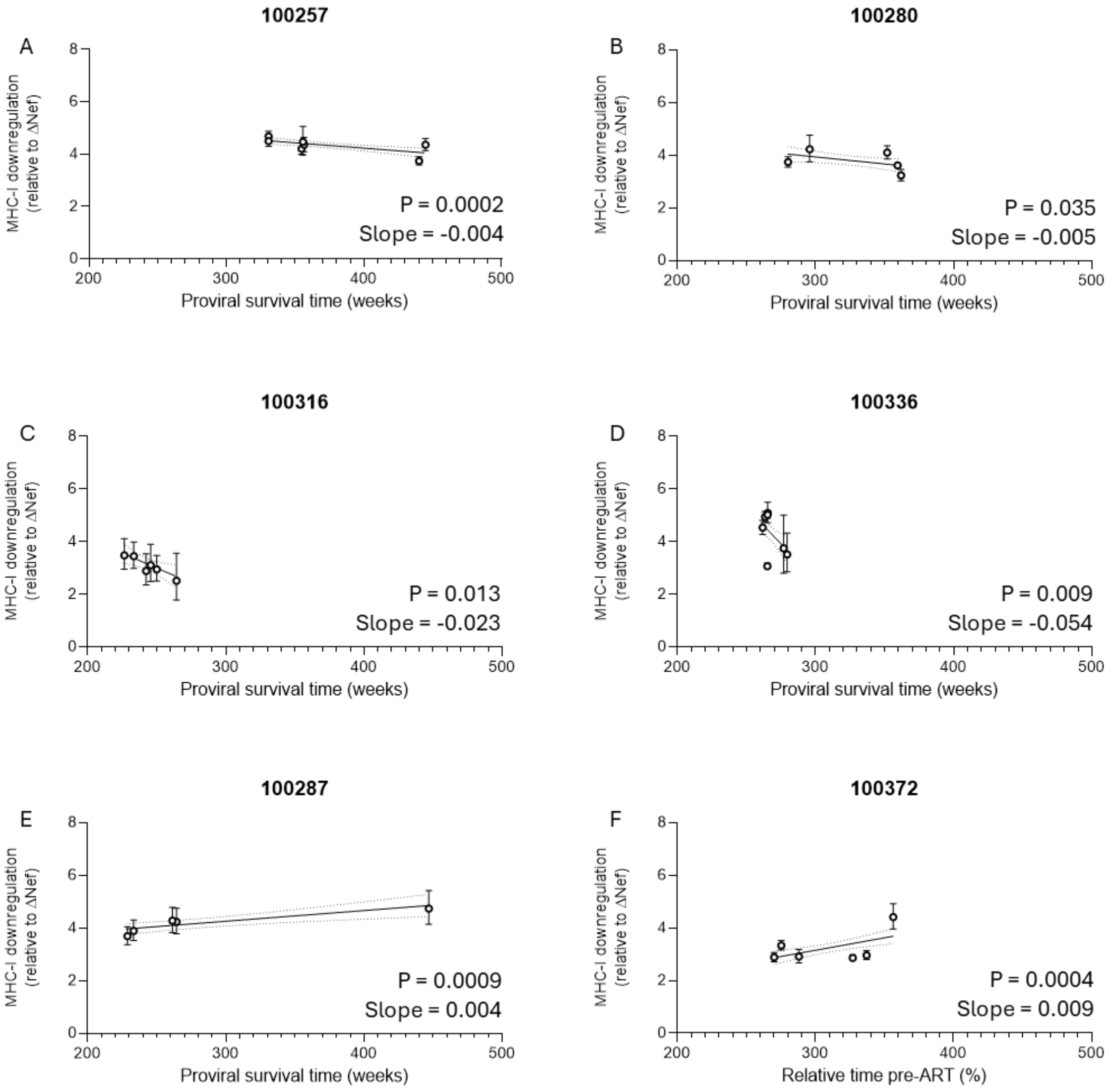

bioRxiv preprint doi: https://doi.org/10.1101/2024.11.01.621615; this version posted November 3, 2024. The copyright holder for this preprint (which was not certified by peer review) is the author/funder. This article is a US Government work. It is not subject to copyright under 17 USC 105 and is also made available for use under a CC0 license.

Figure S4. Individual participants with a significant linear relationship between CD4 downregulation activity and proviral survival time. Each point on the graph represents geometric mean CD4 downregulation for each *nef* clone and the error bars represent the 95% CI. Linear regression best fit lines and 95% CI were plotted for significant linear relationships.

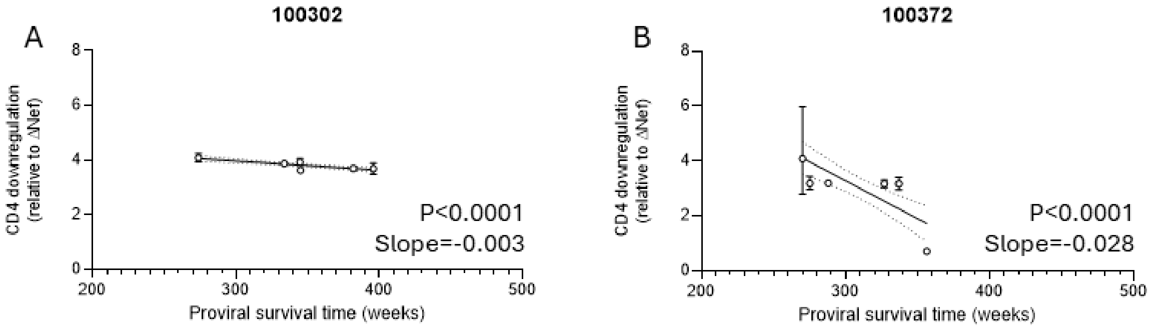

Table S1. Nef subtype prediction for each OGV sequence using the Geno2Pheno Virus Detection and Subtyping tool.

Table S2. Within-participant linear regression analysis for the relationship between CD4 or MHC-I downregulation and estimated proviral survival time.
